# ‘Impairments of the brain’: Global South perspectives on childhood neurodevelopmental disability

**DOI:** 10.1111/dmcn.15253

**Published:** 2022-05-06

**Authors:** Femke Bannink Mbazzi, Elizabeth S. Kawesa

**Affiliations:** ^1^ Social Aspects of Health Medical Research Council/Uganda Virus Research Institute & London School of Hygiene and Tropical Medicine, Uganda Research Unit Entebbe Uganda; ^2^ Faculty of Psychology and Educational Sciences Ghent University Ghent Belgium; ^3^ Global Health and Development London School of Hygiene and Tropical Medicine London UK

## Abstract

The word ‘disability’ does not exist in most African languages. There are words for impairments that describe the ‘missing’ or affected body parts, but no specific words to characterize, for instance, neurodevelopmental conditions. In this narrative review we focus on the different interpretations of neurodevelopmental conditions in Uganda and discuss the importance of cultural concepts and the intersectionality of family‐centred care, poverty, and neocolonialism when working with children with ‘impairments of the brain’. We suggest that these concepts should be taken into consideration in health care service provision and public health awareness campaigns, and the need to rethink how disabled children can be included in a ‘global’ health approach. We explain how applying biomedical categories used in the Global North to diagnose children is only ethical if this information will benefit the child and is accompanied by supportive actions that are relevant to the child and family setting. We argue for the enhancement of community‐based structures of belonging and interdependence that are already in place and strengthening of family and community networks to continue the care and co‐responsibility for children with ‘impairments of the brain’.

**What this paper adds:**

- Cultural concepts, family‐centred care, poverty, and neocolonialism are key in defining disability in the Global South.
- Diagnosis of a neurodevelopmental condition should be accompanied by supportive actions relevant to the child's setting.
- Interventions need to build on family and community‐based structures of belonging and interdependence.
- Health care services and public health awareness campaigns should be made culturally relevant.

## What this paper adds

Illustrated by a case study of a Ugandan boy with an ‘impairment of the brain’, this paper describes the impact of medical models, poverty, and neocolonialism, and the importance of cultural interpretations, communities, and family‐centred care for children with neurodevelopmental disabilities in the Global South

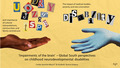

Video Podcast: https://youtu.be/MYORig83rbs

This invited review is commented on by Opai on page 1182 of this issue.

There is a letter to the editor on this invited review by Saloojee et al. on pages 1307–1308 of this issue.

The concepts ‘Global North’ and ‘Global South’ are used to describe groupings of countries using socioeconomic, cultural, historical, and political characteristics. Broadly, the term Global South refers to the regions of South America, Asia, Africa, and Oceania. The use of the term Global South instead of ‘Third World’ or ‘developing countries’ emphasizes the importance of geopolitical relations of power.^1^

In the Global North, concepts of disability are often still explained by medical terminology. The World Health Organization has attempted to shift towards a more holistic model of disability by promoting the use of the International Classification of Functioning, Disability and Health (ICF). The ICF model considers the context in which a disabled individual functions and includes environmental factors such as community and cultural beliefs.^2^

Most countries in the Global South have adopted impairment and disability definitions through medical training, the ratification of the United Nations Convention of the Right of Persons with Disabilities in 2006, commitment to the United Nations Sustainable Development Goals,^3^ as well as the language used in development aid projects. We can question whether these ‘international’ definitions are genuinely international and whether they consider different understandings of disability in different cultures.^4^ The dominance of theories and power from the Global North can easily create generalized and simplified descriptions of disability experiences^5^, ^9^ and can underestimate the importance of cultural concepts and experiences of disability. Disability is a complex, dynamic, and multidimensional concept that has not yet been explored sufficiently in the African context.^10^

Historically, belief systems about the origin of (neurodevelopmental) disabilities in sub‐Saharan Africa involve a mix of biomedical causes, witchcraft, the breaking of taboos, punishment by God, or being the will of God.^11^, ^17^ Various authors have argued that there is a need to learn from indigenous frameworks and take into account local contexts, cultures, and economic and political factors when studying disability in the Global South.^16^, ^18^, ^20^

In this review, we describe a case study which illustrates these factors and propose the use of an Africanized intersectional, theoretical framework. Intersectionality as a theoretical approach has been widely used to discuss sameness and difference in experiences.^21^ Pickens argues that this approach ‘relies on the interrelated nature of identity as formation and lived experience’.^22^ Disability researchers have highlighted the importance of intersectionality and have argued in favour of combining aspects of critical race theory and disability studies into disability critical race studies.^23^, ^25^ In addition to ethnicity, sex, and discrimination, postcolonialism, culture and poverty have been cited as key factors that impact the study of disability in the Global South.^26^

Therefore, in this paper, we look at how a a disabled individual is defined by historical and cultural concepts, what role the individual has in the family and community, and how poverty and neocolonialism are closely linked to the lived experiences, through a narrative case study of a 10‐year‐old Ugandan male with a neurodevelopmental disability. In this paper we refer to Nkrumah's^27^ and Afisi's^28^ definitions of neocolonialism as political, economic, social, and educational subjugation, in which globalization is seen as a new form of neocolonialism that mostly negatively impacts Africa's cultural heritage.

## METHODOLOGY

We reflect on the meaning of disability through the case study of ‘Isma’ (pseudonym), a 10‐year‐old male living in a suburb of Kampala, the capital city of Uganda. Isma and his family are part of the ‘Obuntu bulamu’ study. Obuntu bulamu is an accepted and consistent behaviour that signifies a shared set of values, which promote well‐being, togetherness, and unity. The Obuntu bulamu study explores African concepts of disability and inclusion with an emphasis on belonging, and family and community responsibilities in an attempt to decolonize disability studies in Uganda.^29^ In the first phase of the study, the Obuntu bulamu intervention involved disabled children, parents, teachers, academics, health and rehabilitation workers, and community and district leaders. In the second phase – currently ongoing – this intervention is offered to peer groups of disabled children (*n*=200), their siblings and classmates (*n*=200), their parents (*n*=200), and teachers (*n*=100) in 20 primary schools in the Wakiso and Masaka districts in Central Uganda. The Obuntu bulamu research team consists of community members, participants, researchers, and decision‐makers.

We purposively selected Isma's case from our cohort based on existing data about his family set‐up, socioeconomic background, neurodevelopmental condition, day‐to‐day activities, and home and school community. His case is unique, but in many aspects is also representative of other children in our setting who would be labelled as being ‘on the spectrum’ or having a ‘neurodevelopmental’ condition. Life history and case study research have a long tradition in social science^30^ and Afrocentric research.^31^

The case study information was collected in line with the values of Obuntu bulamu: rather than keeping a scientific distance, we immersed ourselves culturally and socially with Isma and his family, teachers, and community, something which has been described as Afrocentrism by Mkabela,^31^ and Owusu and Mji.^32^ We attempted to ensure that most data collection was conducted by individuals from the research team whom participants could easily relate to. In Isma's case, this was the second author who is a Ugandan clinical psychologist and researcher specialized in the assessment and support of children with neurodevelopmental disabilities. The first author and principal investigator of the project was born in the Netherlands. She is a clinical and educational psychologist, anthropologist, and disability researcher and has been a resident of Uganda since 2003.The team members frequently met with participants at home, in community meetings, and were in touch with each other by phone.

In Isma's case, demographic data about the family was collected from Isma's mother. In‐depth interviews were conducted with Isma's mother, older sibling, a neighbour, and his teacher. Interview questions included general questions about the child and family (e.g. Can you describe your child? Probing for what they enjoy, dislike, behaviour, social skills, etc.), household members (probing for numbers, ages, roles, relationship to, and their interaction with the child), and living conditions. Others were focused on the impairment symptoms (e.g. What are the similarities and differences between the child and other children? When did parents notice that their child behaved differently from other children? What were their concerns?). Questions focused on the response (e.g. How did relatives and friends respond? What support did parents seek and what support did/do they receive?) were asked. Other questions specifically addressed inclusion and participation in daily household tasks, school, and community activities.

The authors visited Isma at home and school on a quarterly basis before the COVID‐19 pandemic and followed up by phone after schools were closed because of the lockdown. Isma responded to the authors' questions by pointing and drawing, his older brother supported him in communicating his ideas with us. Isma also participated in quarterly interactive workshops which were held with all children in the study about their experiences in school between 2017 and 2019.^29.33^ Isma's mother and teacher were part of the development and testing of the Obuntu bulamu training manual between 2017 and 2021.

Interviews were conducted in English and Luganda, the local language commonly spoken in Central Uganda, depending on the preference of the participant. Isma's mother, teacher, and neighbour gave written consent to participate in this study, while Isma and his brother gave their assent. Isma's brother signed the assent form for children and Isma assented by circling yes (smiley face) or no (unhappy face) to questions on a simplified assent form which was read out to him. He pointed at ‘yes’ or ‘no’ to indicate if he had understood and agreed to each point (i.e. he drew pictures and let us take a picture of the drawings to show others). Isma was asked to draw, take photos, and point at people, things, and places he liked and disliked. He was very attentive when instructions were given and would point at the smiley or unhappy faces, or nod to indicate if he had understood.

Interviews were recorded and transcribed verbatim. Visual and observational data were summarized in visual and written reports. Data were reviewed following a thematic approach using framework analysis, a matrix‐based system for organizing, reducing, and synthesizing data^34^ using a codebook developed by the research team. The thematically organized data were reviewed and synthesized. Meaningful themes and quotes were selected to highlight, explain, or describe relevant themes. Identified themes were discussed and checked with participants in follow‐up meetings. The case study summary for this paper was written by the authors and checked with other members of the research team and study participants for validation.

## CASE STUDY

Isma's teacher identified Isma as a disabled child who could potentially benefit from the study. When she was approached to join the study, his mother was open but wondered why the teacher had proposed Isma join the programme: ‘You people [doctors, teachers, and researchers] talk of disability but we are just learning it, it is from what you say that I will say it's a disability.’ His mother had once visited a paediatrician who had mentioned there was something ‘wrong with his brain’ but that there were no medicine or treatment for this condition. His mother had left disappointed to have spent money on the consultation.

Isma is the second child in a family of four children, living with his mother, father, older brother, and a younger brother and sister. Isma's mother runs a small business from home, selling children's clothes and shoes. Isma's father is employed by a small non‐profit organization and is often away; he takes care of the families' financial needs. Isma participates in household activities and chores such as mopping the floor. He is able to wash and dress himself. Before the COVID‐19 outbreak, Isma and his siblings were all in school.

Isma's mother, older sibling, teacher, and neighbour describe Isma as follows.

*Mother:* Isma is different from other children. He loves music, makes sounds, but does not use words. He does not listen to instructions and cannot carry out household chores the way his siblings can. Community members told me to take Isma to the village to meet his relatives, I did, but it did not help. Some thought he was cursed, but I don't think so, this is just the way he was born. At school they say he has impairment of the brain that is why it is hard for him to learn like others. At least he goes to school and has learned to read and write. We would like to move him to Primary One [Isma was in nursery school as per his academic ability in 2020].

*Brother:* Isma likes music, drawing, singing, and climbing trees. He likes to fetch water and play with it. He can get annoyed when he cannot get something that he wants. He pulls you by your hand to take him to what he wants and points at it, he does not talk much, just a few words and sounds, but he understands you when you talk to him. When he is angry or bored he walks away.

*Teacher:* Isma often wanders out of the classroom and roams around the compound. I ask him to engage in activities, but if he does not show interest, I just let him be. Sometimes I manage to call him back into the classroom. When given pencil and paper, Isma does sit down and starts drawing detailed images of cars. He repeats sounds but does not read words nor speak sentences. I don't think Isma can complete primary school like other children, but it is good for him to be here and learn with others.

*Neighbour:* In the community Isma mostly moves around with his brother. They fetch water and climb trees and pick mangoes if it is mango season. Isma enjoys playing with water, which annoys some of the community members (some feel he wastes water). He does not play football like the other boys. Sometimes Isma moves around on his own, he knows his way around, though neighbours will walk him back home if they feel he is lost or should return because it is late. We call him the child that does not speak but hears some things.

During home and school visits, which took place quarterly during the study, Isma was observed to enjoy repetitively drawing items that he observed around him. Before the COVID‐19‐related school closure, Isma was observed to engage in classroom activities when given very clear, step‐by‐step instructions by his teacher. He made sounds to communicate to others if he did not want to do something or if he needed something. He would also walk up to a person and point or pull them to the item he wanted. Isma was observed to communicate through pointing and would increase the volume and heighten the tone of his voice when not understood or not given what he asked for. Isma also mimics some people like preachers and teachers. Isma loves to sing and has been observed to repeatedly sing songs he enjoys. During the COVID‐19 lockdown, Isma was observed to draw more at home. He would get frustrated, make loud sounds, and move around, especially when the paper he used for drawing ran out. His mother noticed how much he missed school, she explained Isma cried a lot more as he missed the things that keep him calm: routine activities, new challenges in learning, and the possibility to draw on paper. She and her husband try their best to buy paper and ensure he is included in home activities as much as possible. Isma started using single words to ask for items during the lockdown period and would communicate by singing his favourite songs and display his drawings to his family and the authors when visited.

**Figure 1:**
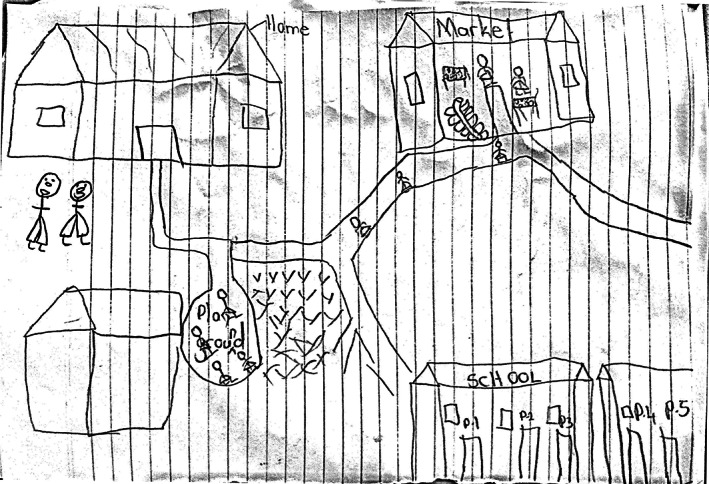
Isma's drawing of spaces

In his living spaces drawing in October 2021 (Figure 1), Isma's brother supported Isma in drawing the spaces of his life. His brother drew their home. Isma was asked what he would like to add; he added a playground that is close to their home. His brother commented that they no longer go to the playground because of COVID‐19, and that they play from home more now. Isma added the market; his brother explained: ‘This is where we buy some vegetables if mum has not gone to the big market’. Isma and his brother drew his school, which is within walking distance. At the time of drawing, Isma had not gone to school for 20 months due to the COVID‐19 lockdown in Uganda. His mother mentioned that Isma loves going to school and will sometimes wake up, dress up in his school uniform, and, if not monitored, walk to his school (even if closed). When all spaces were drawn and Isma was asked if there was anything to add, his younger brother added two people which he named ‘Isma and I', which Isma seemed to approve of.

The descriptions given by the various individuals who are part of Isma's life show important historical and cultural aspects of defining disability, the importance of belonging and relying on family and community, and, implicitly, the impact of poverty. We discuss the following themes based on the case study: history and culture, family and belonging, and poverty and access to health care.

### History and culture

In Isma's story we see the historical concept of ‘being cursed’ and the importance of seeing the extended family about it to enable a solution or way forward. Isma's mother followed the advice of the family even if she did not think he was ‘cursed’ and said it did not help in changing Isma's condition. She respected and put what others suggested above her own doubt on whether anything would change, emphasizing interconnectedness and the importance of the community over the individual. With this emphasis on the community, the need to ‘diagnose’ and ‘treat’ Isma as an individual is moved to the background. The important matter is his being recognized and included in the community and the community's support in looking after him, even if the ‘curse’ was not taken away and Isma's condition did not change.

Looking at Isma's mother, sibling, teacher, and the neighbour's descriptions from a Global North biomedical perspective, they all explain Isma as having ‘typical symptoms’ of a child ‘on the spectrum’: he is different; he makes sounds, but points rather than using words; he is fixated on playing with water and making very detailed drawings; he gets upset when things do not go his way; he seems to be in a different world and is disengaged from other students and what goes on at school; he does not play football with other children; he moves around on his own or with his brother. To find words which describe this medically but not in medical terms, the doctors and teachers have given Isma's mother a description of the medical diagnosis: Isma has ‘an impairment of the brain’, something is wrong with this part of his body and there is nothing that can be done about it.

What Isma's family and community members describe are the things he cannot do that culturally he is expected to do. For example, he is expected to greet others he meets in passing – Isma does not; he is expected to help out in the house – Isma rarely does so; in school, he should sit in class and learn – Isma follows his own interests instead. However, they also describe his likes and dislikes, the things he can and does participate in and has a preference for, such as mopping the floor, and the way they look after him together.

### Family and belonging

Isma's mother went to see his father's family. It is her responsibility to visit them to consult them about Isma and other issues. In turn, his relatives have a responsibility for the care and upbringing of Isma. The descriptions of Isma's life emphasize the importance of reciprocity and participation in community. Isma's schooling is paid for by his family. His teacher feels it is important for him to go to school so that he is with other children. His mother has the expectation that he will learn to read but is aware he cannot learn ‘like others’. She hopes that Isma obtains basic reading and counting skills and can participate in home and agricultural activities.

In school, Isma has a peer who looks out for him and encourages him to participate in class. He helps Isma by telling other children to leave him alone at times. Because of the COVID‐19‐related school closure in Uganda, Isma was home for 22 months. Isma's brother kept an eye on him. His mother noticed how much Isma has missed going to school.

As Isma is ‘the child who does not talk but hears some things’, community members do not expect him to speak, but they still talk to him and engage him in activities where possible. If needed, they will bring him home if he is out alone. Isma's family and community are key in supporting Isma to learn and offer him a sense of belonging. They look out for him and consider him part of their community.

### Poverty and access to health care

The lack of reference to disability information and health care, let alone specialized interventions, in Isma's narrative illustrate the non‐existence of these services in his community. At school he might draw ‘when given pencil and paper’, not because his teacher does not want to give him the paper, but because his school does not have sufficient supplies to allow Isma to draw every day. The comments of community members and his brother about the dislike of Isma playing with water refers to the importance of water and its scarcity in many communities. During the COVID‐19 pandemic, Isma stayed home. Like many other parents in Uganda, Isma's mother was unsure how to go about home learning. She could not afford a tutor or online learning and could not reach relatives and other people in her network because of transport bans during lockdown.

## DISCUSSION

In this paper we have discussed the importance of historical and cultural concepts including neocolonialism, intersectionality of family‐centred care, and poverty, when working with children with ‘impairments of the brain’ in Uganda.

### Disability definitions in the Global South

Disability studies aim to explore the meaning of disability in society.^35^ As each society has its own history and social fabric, disability is also affected by history, culture, and context. In East Africa, disability is often described as an ‘affected or missing’ body part or function,^36^, ^37^ and an inability to participate in household tasks and community activities.^38^, ^39^ From a Global North perspective, we may define these as ‘social’ implications; however, in critical disability studies^40^ and Afrocentricity^32^ we consider these descriptions from the viewpoint of social embodiment^41^ and indigenous understanding of what impairment is and how this evolves in a specific cultural setting.

Neurodevelopmental disabilities in the Global South do not fit into one single description and, therefore, cannot be treated in the same way as in the Global North.

Disability cannot be simplified or generalized. As Grech argues ‘disability across cultures, contexts, spaces, and places can hardly be encapsulated in all embracing static models, goals, or strategies’.^42^ Children with neurodevelopmental disabilities or children with ‘impairments of the brain’ are not a homogenous group, yet disabled individuals are often referred to as a uniform group of people.^43^ Similarly ‘African’ studies are frequently grouped as if experiences in a particular context can be generalized to the entire continent.^44^ As Julie Livingston in her book *Debility and the Moral Imagination in Botswana* ^45^ and Nirmala Everelles in *Disability and Difference in Global Contexts* ^46^ make clear, definitions of disability cannot and should not be moved carelessly across transnational borders. Our case study is, therefore, not meant to be a representation of children with neurodevelopmental disabilities in the Global South, but an illustration of how important sensitivity to historical and cultural concepts is.

### Historical and cultural concepts of disability

The historical beliefs about disability and their causes have been influenced by colonialism and neocolonialism. Most concepts of disability in the Global South and how to ‘treat’ disabled individuals are imported from the Global North.^6^, ^47^ Colonial powers had an interest in portraying local practices as ‘backward’, as the ‘savage’ needed to be ‘civilized’.^48^ Missionaries and health professionals were closely linked to colonial powers, and were often brought in to ‘treat’ disabled individuals.^42^ Post colonialisation, former colonists still had a huge say in the internal affairs of the nations, which impacted the way disability was, and still is, defined.^45^ Neocolonialism continues to influence the knowledge transmitted, the curricula, references, and pedagogy in academic institutions in the Global South,^48^ often keeping imported concepts and practices alive and unquestioned. The origins of ‘development’ and related projects and approaches used to address disability in most sub‐Saharan African countries, such as community‐based rehabilitation or community‐based inclusive development, are closely related to neocolonialism.^42^ Definitions of impairments and disability in our context thus consist of a variety of diagnostic labels from the Global North, indigenous descriptions of impairment and disability, and a mix of both. In our study the description ‘impairments of the brain’ was used by several parents; it is constructed through the interaction of families' descriptions of neurodevelopmental disability, and the biomedical explanations given by health workers. This terminology shows the intersection of imported biomedical and disability terminologies and indigenous understandings of the impairment.

### Families and communities

Traditionally, and still today, the family is a very strong cultural institution in most African countries.^26^ Children are considered members of an extended rather than nuclear family, and relatives beyond the nuclear family are responsible for their shelter, clothing, food, and tuition fees.^49^, ^51^ Involving family members in the diagnosis and care of children with neurodevelopmental disorders is, therefore, key. Children may not necessarily be served by programmes that focus on ‘rehabilitating’ the individual child to an ‘independent’ adult in this context. Furthermore, informal and traditional forms of care and participation are important yet are often forgotten.^10^ Isma is supported by his community. The community finds different ways of including and communicating with him, because there is a history and culture of communal responsibility to take care of ‘our’ children, including children ‘who do not speak but hear some things’. Reflecting on disability from an African perspective can help move us beyond a narrative of deficit and stigma, to emphasize the capabilities of communities and society.^10^

### Poverty and health care

Grech has emphasized the importance of placing the relationship between poverty and disability in context. He argues that the relationship between poverty, livelihoods, and disability is complex, non‐linear, and non‐circular.^52^ Disability affects human capital (access to education, health care, work) as well as social, financial, physical, and natural capital.^53^ Most families of disabled children in sub‐Saharan Africa depend on natural resources and manual labour for their livelihood and live in countries in which a welfare system is absent.^52^ Disability can reduce the chances of employment and education, and increase financial expenses and caring responsibilities.^35^, ^54^, ^55^

Disabled individuals are commonly excluded from health care^42^, ^56^ and specialized care for children with neurodevelopmental disabilities in the Global South is limited.^57^, ^58^ This can partly be explained by the scope and funding of public health programmes in the Global South which focus on the needs of the general population, thereby excluding the requirements of individuals with particular needs.^59^ Social and political arrangements influence health and access to health care.^59^, ^60^ Even if disability‐specific interventions are available, they can themselves become a barrier if undertaken without taking the context into consideration.^48^ The vulnerability of disabled individuals to disasters, climate change, and health issues, such as the COVID‐19 pandemic, has been documented and is likely to increase as inequality increases.^59^, ^61^

As much as we agree that access to health care and education should be for all, the reality for many families in the Global South is that specialized care and inclusive education for children with disabilities, as known in the Global North, is unlikely to be attained soon. Policy implementation challenges are common in Africa^62^ and the lack of acknowledgement of local practices in policy application is a great concern.^43^ It is questionable if the inclusive health and education policies and the imported ‘aid’ programmes from the Global North will provide the support that families require. A good example of this is the importation of new technologies, which can be relevant and bring social change, but are often ill‐fitted to the society they are donated or sold to.^63^ Global North policies disregard indigenous practices, often emphasizing independence and self‐presentation of the individual over the collective good, which causes tension in countries in which the ‘we’ supersedes the ‘I'.^10^, ^32^

Social indigenous practices can build solidarity and support and inclusion of disabled individuals and provide lifelong support in context,^59^ even if poverty and inequality are still prevalent. These practices may challenge ‘developmental’ ideas of rehabilitation, modern technology, and the importance of ‘independence’. They require rethinking the use and meaning of the term ‘neurodevelopmental disabilities’ with individuals with ‘impairments of the brain’ to better understand their needs.

## CONCLUSION AND RECOMMENDATIONS

Medical categories and labels can be helpful for health workers and service providers to know ‘what to do’. For Isma's mother and many other parents, the label might help validate the fact that there is something ‘different’ about their child, but it is questionable whether knowing and naming this is useful if there is no specialized, cultural relevant, and accessible care and support in the community they live in that relates to the diagnosis. Grech has argued that rights and policies mean little in practice for the poorest.^42^ Similarly, an imported diagnosis means little if there is no follow‐up. Providing a diagnosis without being able to offer follow‐up raises an ethical question. In her book *Black Madness,* Pickens describes how she ‘keeps a tension between psychosocial definitions of madness (without attributing causality) and biomedical definitions (without attributing authority)’.^22^ We argue to consider cultural definitions and knowledge in addition to biomedical definitions of ‘impairments of the brain’ in diagnosis, health care, and raising awareness about neurodevelopmental disorders in the Global South.

More research is needed to document the needs of individuals with neurodevelopmental disorders in the Global South, to inform global health actors designing ‘interventions’ for children ‘diagnosed’ with neurodevelopmental disabilities. They are often spoken for by others,^18^ as they communicate in a way that researchers are unfamiliar with or do not have ‘methods’ and ‘tools’ for.^33^, ^34^ Alternative, culturally relevant research methods need to be explored to ensure the views of children with ‘impairments of the brain’ are documented and used to inform treatment and care. Children with disabilities together with their families and communities can and must be included and consulted in designing a ‘global’ health approach as well as specific ‘interventions’. Instead of importing community‐based inclusive development interventions from the Global North that are ‘culturally adjusted’ by a team of global health experts, we argue that community‐based structures of belonging and interdependence that are already in place should be built on, and family and community networks strengthened to continue the care of and co‐responsibility for children with ‘impairments of the brain’.

## Data Availability

The data that support the findings of this study are available from the corresponding author upon reasonable request.
